# LSTM-SAGDTA: Predicting Drug-target Binding Affinity with an Attention Graph Neural Network and LSTM Approach

**DOI:** 10.2174/0113816128282837240130102817

**Published:** 2024-02-27

**Authors:** Wenjing Qiu, Qianle Liang, Liyi Yu, Xuan Xiao, Wangren Qiu, Weizhong Lin

**Affiliations:** 1 School of Information Engineering, Jingdezhen Ceramic University, Jingdezhen 333000, China

**Keywords:** LSTM-SAGDTA, high-throughput screening, graph attention networks, binding affinity, state-of-the-art methods, DTA predictor

## Abstract

**Introduction:**

Drug development is a challenging and costly process, yet it plays a crucial role in improving healthcare outcomes. Drug development requires extensive research and testing to meet the demands for economic efficiency, cures, and pain relief.

**Methods:**

Drug development is a vital research area that necessitates innovation and collaboration to achieve significant breakthroughs. Computer-aided drug design provides a promising avenue for drug discovery and development by reducing costs and improving the efficiency of drug design and testing.

**Results:**

In this study, a novel model, namely LSTM-SAGDTA, capable of accurately predicting drug-target binding affinity, was developed. We employed SeqVec for characterizing the protein and utilized the graph neural networks to capture information on drug molecules. By introducing self-attentive graph pooling, the model achieved greater accuracy and efficiency in predicting drug-target binding affinity.

**Conclusion:**

Moreover, LSTM-SAGDTA obtained superior accuracy over current state-of-the-art methods only by using less training time. The results of experiments suggest that this method represents a high-precision solution for the DTA predictor.

## 1 INTRODUCTION

Drug development is a challenging and costly process [1, 2], yet it plays a crucial role in improving healthcare outcomes. Drug development requires extensive research and testing to meet the demands for economic efficiency, cures, and pain relief. Target identification and validation are among the most critical steps in developing new drugs or repurposing existing ones. Traditionally, experimental approaches to drug repositioning involve high-throughput assays that test a library of approved drug compounds against biological targets of interest. However, high-throughput screening (HTS) is an expensive and time-consuming component of the drug discovery process [3]. The vast number of potential drug compounds and targets makes a brute-force search impractical, with millions of options to consider [4]. However, recent technological advancements have enabled faster and more cost-effective drug development through Computer-Aided Drug Design (CADD) [5, 6]. CADD employs computational methods and algorithms to simulate molecular interactions between drugs and biological targets, allowing researchers to narrow down the search space for new drugs, predict drug efficacy and toxicity, and improve drug safety profiles. Compared to wet-lab experiments, CADD offers higher efficiency and success rates, particularly in repurposing known drugs [7, 8].

In modern drug design, the search for matching drug molecules and proteins, also known as Drug-Target Interactions (DTI), is a crucial aspect of computer-aided design. The Drug-Target binding Affinity (DTA) provides information about the strength of DTI, making it an important criterion for selecting candidate compounds in drug development [9].

For proteins with structural and site information, drug molecules can be directly evaluated for binding affinity through molecular docking by using molecular simulations [10] and molecular docking [11]. Methodologies, like DOCK [12], AutoDock [13], and GOLD [14], provide computational models that predict how tightly the drug binds to the protein surface. However, many proteins still lack known structures, and even their detailed structural information may not be obtained after spending a lot of time on homology modeling.

To tackle these challenges, two effective machine learning methods based on similarity have been proposed: KronRLS [15] and SimBoost [16]. Similarity-based methods assume that similar drugs tend to interact with similar targets, and similar targets interact with similar drugs [17]. KronRLS defines the similarity score of a drug-target pair by taking the Kronecker product of the similarity matrix, which is given as the paradigm of the prediction model and associated with a symmetric similarity measure. SimBoost, on the other hand, employs a gradient-boosting machine with novel feature engineering to extract new features from drug, target, and drug-target pairs in the training dataset.

Although the previously mentioned methods have shown good performance in predicting DTA, they heavily rely on chemical insights or expert experience, and manual feature extraction requires significant biological prior knowledge [18]. To overcome these challenges, automatic feature extraction methods, such as auto-encoders and converters developed using deep learning, have shown great promise.

With the development of artificial intelligence, deep learning methods provide a more equitable and reasonable solution for DTA prediction. DeepDTA [19], presented as the first sequence-based deep learning model for predicting DTA, is an end-to-end convolutional neural network (CNN)-based model that predicts affinity scores directly without requiring feature engineering. Features are automatically captured by the backpropagation of a multilayer CNN. To further improve performance, DeepDTA collected previous data and constructed two benchmark datasets, Davis [1] and Kiba [20]. WideDTA [21] is an extension of DeepDTA that utilizes Ligand Max Common Substructure (LMCS) [22] and higher-level features from PROSITE's Protein Structural Domain Mapping or Motifs (PDM) [23]. Another deep learning model, DeepAffinity [24], uses one-hot encoding to encode drug SMILES [25] (Simplified Molecular Input Line Entry Specification) and encodes proteins into sequences with structural properties containing detailed structural information and higher resolution. Subsequently, drug SMILES and protein structural sequences are encoded into embedded representations using seq2seq [26]. Seq2seq, a recurrent neural network (RNN) autoencoder model, maps the original sequences to vectors learned in an unsupervised mode to capture the dependencies in SMILES or protein residue sequences. GANsDTA [27] represented drug and protein features by two generative adversarial networks (GAN) for prediction. In BiComp-DTA [28], a three-layer fully-connected neural network was employed to extract the protein feature, while a network with two CNN layers, followed by a separable CNN layer, was used to learn the drug representation.

In recent years, there has been an increasing application of graph neural networks (GNN) in extracting inherent features of proteins [29-33]. In the field of DTA, GraphDTA [34] first introduced GNN into DTA prediction. It extracted drug features using graph convolutional networks (GCN) [35], graph attention networks (GAT) [36], and graph isomorphic networks (GIN) [37]. DeepGS [38] utilized GNN to express drug features by formulating amino acids and atoms by Prot2Vec [39] and Smi2Vec [40].

Self-attention has been particularly effective in enhancing the performance of one-dimensional or structure-based representations by helping the network concentrate on the most relevant parts of the input while also reducing the loss of implicit information [36, 41, 42]. For instance, AttentionDTA [43] adds an extra attention block after the two branches for drugs and proteins, which allows the network to learn features weighted according to the attention score before feeding them to the fully connected classification layer. In addition, Lim *et al.* [44] proposed a distance-aware attention algorithm that captures the most relevant intermolecular interactions in complex structural information. Self-attention graph pooling (SAGPool), which utilizes graph convolution to obtain self-attention scores, has achieved state-of-the-art performance in several graph learning tasks, as reported in recent studies by Lee *et al.* [45-48].

In this study, we proposed a novel DTA prediction model that utilizes an LSTM-based language model to encode protein sequences and a simplified network structure combined with a self-attention graph pooling approach to learning molecular graph representation. We evaluated two different architectures, global pooling (GlobPool) and hierarchical pooling (HierPool). Moreover, our model presents two major innovations that improve upon existing models.

## 2 MATERIALS AND METHODS

### 2.1 Materials

To ensure a fair and comparable evaluation, we evaluated our approach on two widely-used benchmark datasets, namely Davis and KIBA, which were initially introduced by DeepDTA for predicting the binding affinity between drugs and target proteins. Each dataset contained a large number of binding entities, with each entity having a pair of a drug molecule and a target protein.

Furthermore, we conducted testing and evaluation of our model's generalization ability on the recently updated publicly available dataset, PDBbind [49, 50], which consists of experimentally measured binding affinity data for drug-target complexes stored in the Protein Data Bank (PDB) [51]. More information about these datasets is provided in Table **1**.

According to the statistics reported in our study, the Smith-Waterman (S-W) [52] similarity between proteins was at most 60% for 99% of the protein pairs in the KIBA dataset. Similarly, 92% of the protein pairs in the Davis dataset had a maximum target similarity of 60%. These statistics suggest that both datasets are non-redundant.

The Davis dataset comprises a total of 30,056 protein-drug pairs with binding affinity values, involving 442 unique proteins and 68 unique compounds. The KIBA dataset consists of 229 unique proteins and 2,111 unique drugs, containing 118,254 protein-drug pairs, which are collected from ChEMBL [53-58] and STITCH [59-63].

For the PDBbind dataset, we selected the refined set from the 2020 version due to its superior data quality and larger size. This particular set includes Ki and Kd [64] values, which have been transformed into log-scale (pKi and pKd values). It contains binding affinity data for 4295 drugs and 1606 protein targets. To ensure data consistency, redundancies in drugs with multiple sequences in SMILES format were eliminated. As a result, the final utilized set encompasses binding affinity values for 4231 drugs and 1606 protein targets.

### 2.2 Model Architecture

In this study, we proposed a novel method for predicting DTA called LSTM-SAGDTA, which takes drug-protein target pairs as input and generates the corresponding affinity value as output. We leveraged RDKit [65], an open-source chemical software, to generate drug SMILES molecular symbols as drug molecular maps, where atoms serve as nodes and bonds as edges. Meanwhile, we used SeqVec [66], a language model based on LSTM features, to characterize protein sequences with long and short-term dependent information. To simplify the network structure, multiple layers were replaced with a single layer, and only two graph neural network layers, GCN and GAT, were utilized to learn the drug representation and a single 1D CNN layer to learn the representation of proteins [67]. To enhance DTA prediction accuracy, we employed a new self-attentive graph pooling method that selectively aggregates important information rather than coarse pooling. Furthermore, we implemented and evaluated both global pooling (GlobPool) and hierarchical pooling (HierPool) architectures for self-attentive graph pooling. The overall architecture of the model is depicted in Fig. (**1**).

### 2.3 Protein Representation

SeqVec, a method that utilizes the deep bidirectional language model ELMo [68], has demonstrated success in predicting protein structure and function by representing protein sequences as continuous vectors. The ELMo model consists of a context-insensitive CharCNN [69] and a two-layer bidirectional LSTM that captures contextual information from the surrounding words. In this study, the sequence was cut or padded to a fixed-length sequence of 640 amino acids. Next, the sequence was embedded into 1024-dimensional vectors by SeqVec. Then, the LSTM [70] and the 1D convolutional neural network were used to learn different levels of hidden features and apply a maximum pooling layer to obtain a representation vector of the input protein sequence.

### 2.4 Drug Representation

The open-source chemical software RDKit was utilized to convert drug SMILES into a molecular graph and extract the atomic features. To capture the complete chemical and binding properties of small molecules, it is important to incorporate features that describe atomic nodes. In our study, we used the same feature selection for atoms as in GraphDTA and adopted a set of atomic features from DeepChem [71], as outlined in Table **2**. By characterizing atomic nodes using these features, we can more accurately represent the chemical and binding properties of small molecules.

We then adopted single-layer GAT and single-layer GCN network structures to learn the representation of drug molecule graphs. To aggregate important information, a self-attention graph pooling layer was employed. There are two pooling strategies: hierarchical graph attention pooling and global self-attention graph pooling. Hierarchical graph attention pooling involves two convolution blocks, each consisting of a graph convolution layer and a SAGPooling layer. The convolution results of each layer are pooled and read out in layers, and the two read-out results are summed and finally passed to the fully connected layer to obtain the final drug representation. In contrast, global self-attention graph pooling involves linking two graph convolution layers in series, combining the two outputs, and sending them to the SAGPooling layer for global pooling. The node features are aggregated in the readout layer after the pooling layer and finally passed to the fully connected layer to obtain the drug molecule representation. A graph convolution was used to obtain self-attentive scores. Therefore, the pooling results were found to be based on graph features and retained topological information.

### 2.5 Evaluation Method and Performance Metrics

To validate the effectiveness of models, the five-fold cross-validation was adopted. The metrics utilized to evaluate the performance of models are (i) CI: consistency index and (ii) MSE: mean square error.

CI, proposed by GÖnen and Heller [72], was used to measure the difference between the predicted and actual values of the model, with higher values indicating better performance. The CI is defined as follows:


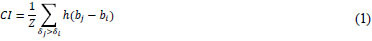


Where *b_i_* is the predicted value of the smaller affinity *δ_i_*, *b_j_* is the predicted value of the larger affinity *δ_j_*, h(x) is the step function, and Z is the normalized hyperparameter. Generally, the step function h(x) is defined as follows:


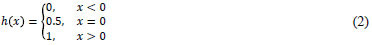


In addition to CI, MSE was also used to assess the difference between predicted and actual values. It is a statistical measure that directly evaluates error. Assuming that the estimate has n samples and n corresponding true values, the MSE is expressed as the expected value of the squared loss, with smaller values indicating better performance. The equation is as follows:


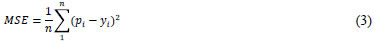


Where, *ρ_i_* is the predicted value and *y_i_* is the actual value; a smaller MSE means that the predicted value of each sample is closer to the true value.

## 3 RESULTS AND DISCUSSION

### 3.1 Model Evaluation Result

We performed a five-fold cross-validation to evaluate the model performance on both the Davis and Kiba training datasets. LSTM-SAGDTA with HierPool achieved remarkably good results, as mentioned in Table **3**, which presents the metrics for each fold. The model achieved an MSE score of 0.212 and a CI score of 0.895 on the Davis training dataset and an MSE score of 0.141 and a CI score of 0.895 on the Kiba training dataset. In each fold, there was a slight deviation in the MSE and CI metrics. These outcomes indicated that LSTM-SAGDTA has robust performance and good generalization capability. Therefore, our proposed method shows great promise and warrants further investigation.

### 3.2 Comparing LSTM-SAGDTA to Baseline Models

We conducted a comprehensive evaluation of our method on the independent test dataset of Davis and Kiba, respectively, and compared it with seven other existing baseline methods, namely KronRLS [15], SimBoost [16], DeepDTA [19], WideDTA [21], GraphDTA [34], DeepGS [38], AttentionDTA [43], and BiComp-DTA [28]. We analyzed the results of the experiments and summarized them in Table **4**, using either MSE or CI as the evaluation metric. Our methods outperformed all other models in terms of both metrics across both datasets, demonstrating their effectiveness in comparison to the existing state-of-the-art methods. The results of our experiments conclusively establish the superiority of our proposed method.

**Fig. (2):**
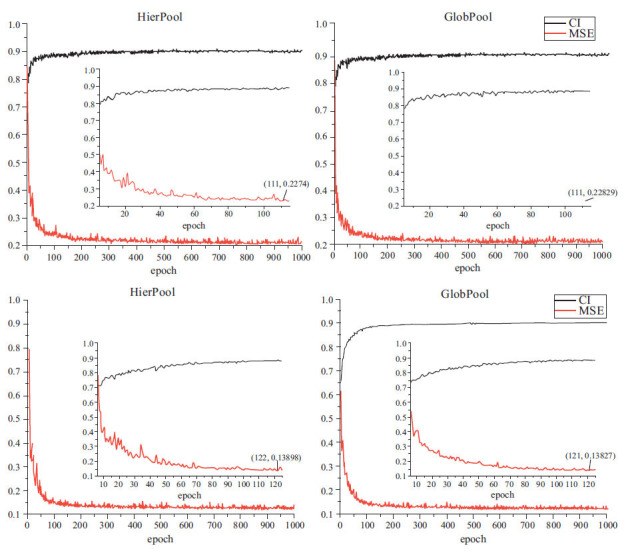
CI and MSE values of LSTM-SAGDTA for each epoch on the Davis and Kiba datasets.

Table **4** presents the results of each model on the Davis dataset, with our models’ results shown in bold. Results are rounded to 3 decimal places. Our proposed method outperforms all the baseline methods in terms of both MSE and CI on the Davis dataset, including a one-dimensional representation of sequences and other graph-based methods. The hierarchical pooling strategy achieves the best performance with an optimal MSE of 0.206 and CI of 0.903 among the two pooling architectures. This represents an improvement of over 2 percentage points in terms of MSE compared to GraphDTA. Moreover, our method outperformed even the best variant of GraphDTA after just 111 epochs, with an MSE result of 0.227 and a CI of 0.891. While GraphDTA achieves the same performance as our model after 1000 epochs (Fig. **2**). These results demonstrated that our novel approach has great potential for improving DTA predictions.

The global pooling strategy also performed well on the Davis dataset, albeit slightly worse than the hierarchical pooling strategy. Compared to the other baseline models, the global pooling strategy achieved better results, with an optimal MSE of 0.208 and a CI of 0.904. It was able to reduce the MSE by more than 2 percentage points compared to GraphDTA. At 111 epochs, the MSE was 0.228, and the CI was 0.888, while at 153 epochs, it improved by one score point over GraphDTA, with an MSE of 0.219 and a CI of 0.891. These results demonstrated the efficacy and robustness of our proposed model for DTA prediction.

Table **4** also presents the results obtained by different methods on the Kiba dataset. Consistent with the findings from the Davis dataset, the models presented in this study outperformed existing approaches across all evaluation metrics. However, on the Kiba dataset, the global pooling strategy yielded stronger performance than the hierarchical pooling strategy. In particular, the global pooling strategy attained an MSE of 0.122 and a CI of 0.902, translating into an improvement of nearly 2 percentage points over the baseline model GraphDTA. Furthermore, the global pooling strategy exhibited rapid convergence behavior, as shown in Fig. (**2**). By only 119 epochs, the global pooling strategy surpassed the performance of GraphDTA, with an MSE of 0.137 and a CI of 0.886, whereas the hierarchical pooling strategy reached this level at 122 epochs with an MSE of 0.138 and a CI of 0.886. These results confirmed the merit and adaptability of the proposed models for DTA prediction tasks.

To further demonstrate the strong generalization and robustness of our model, we conducted training and testing evaluations on the recently updated open-access dataset, PDBbind. We compared it with four baseline models: DeepDTA [19], GraphDTA [34], FusionDTA [73], and BiComp-DTA [28]. The experimental results, which are presented and summarized in Table **5**, were analyzed using MSE or CI as evaluation metrics. The results indicated that our method outperforms all other models on the PDBbind dataset, as evidenced by superior performance on both metrics. The outcome confirms the broader applicability and practicality of our proposed approach.

Our experimental evaluations have established the superiority of the proposed model in terms of its predictive and generalizability abilities across the Davis, Kiba, and PDBbind datasets. When compared against state-of-the-art benchmark models, our model consistently outperformed them across various evaluation metrics. Both the global and hierarchical pooling strategies showed promising results, emphasizing the significance of selecting an appropriate pooling technique depending on the given dataset. This observation underscores the necessity for flexible neural architectures customized to individual datasets.

### 3.3 Comparison of LSTM-SAGDTA in Terms of Various Network Structures

To assess the impact of design choices of the proposed model, we conducted a control experiment using alternative network configurations. The control experiment maintained the same network parameter variables and drug and protein-coding methods as the proposed model but altered the network architecture. Specifically, two new network structures were tested for comparison: a single-layer GCN combined with a single-layer GAT (GAT-GCN) and a three-layer GCN (3GCN). The results of these comparisons are provided in Table **6**.

### 3.4 Comparison of Pooling Strategy Performance

Different pooling methods have unique properties within a model architecture, enabling the model to emphasize various parts of the intermediate sequence, which, in turn, influences the update of layer parameters with different gradients [74]. Max-pooling and mean-pooling are commonly employed techniques that use maximum or average values to aggregate feature maps into tokens. However, these methods may not fully capture the underlying chemical patterns present in molecules. Therefore, we employed a novel graph pooling method based on self-attentiveness, known as Self-Attentive Graph Pooling (SAGPool) [45]. Unlike traditional pooling methods, SAGPool uses a self-attentive mechanism to selectively focus on relevant information in the input sequence. This allows the model to learn more complex and efficient feature representations for prediction tasks.

To optimize the performance of our models, we investigated several pooling strategies. Instead of using the Sag-Pool layer, as shown in Figs. (**1b** and **1c**), we substituted it with Max-pooling or Mean-pooling, respectively. By comparing the results obtained from these alternatives on the Davis and Kiba datasets, we aimed to identify the best pooling strategy for improving model accuracy. The corresponding evaluation results are summarized in Table **7**. We discovered that Sag-Pool achieved the best performance. This suggests that Sag-Pool captures essential information about drug molecules and leads to superior model performance.

## CONCLUSION

Based on the results, it is evident that the simplified network architecture proposed in this paper, which integrates a single-layer GAT and single-layer GCN, outperforms the conventional multi-layer GCN network model. This implies that the number of layers is a critical parameter that impacts the performance of deep learning models. While it is commonly believed that increasing the number of GNN layers can capture more information from node and edge features, too many layers may lead to poor performance due to gradient disappearance and over-smoothing. Therefore, more complex network structures with multiple layers do not necessarily guarantee improved model performance, and simple models can achieve excellent results in practice.

By comparing the two pooling strategies, it can be observed that on the Davis dataset, the hierarchical pooling strategy outperformed the global pooling strategy for both the GAT-GCN and 3GCN architectures. However, on the KIBA dataset, the global pooling strategy yielded better results than the hierarchical pooling strategy for both network structures. These findings underscore the importance of data-driven models and the significance of selecting the appropriate pooling architecture for different datasets. Ultimately, the choice of network architecture, the number of layers, and the pooling strategy can significantly impact the performance of a deep learning model. It is worth noting that simpler models can often achieve excellent results. Therefore, it is crucial to consider a range of factors when designing and evaluating deep learning models.

## Figures and Tables

**Fig. (1):**
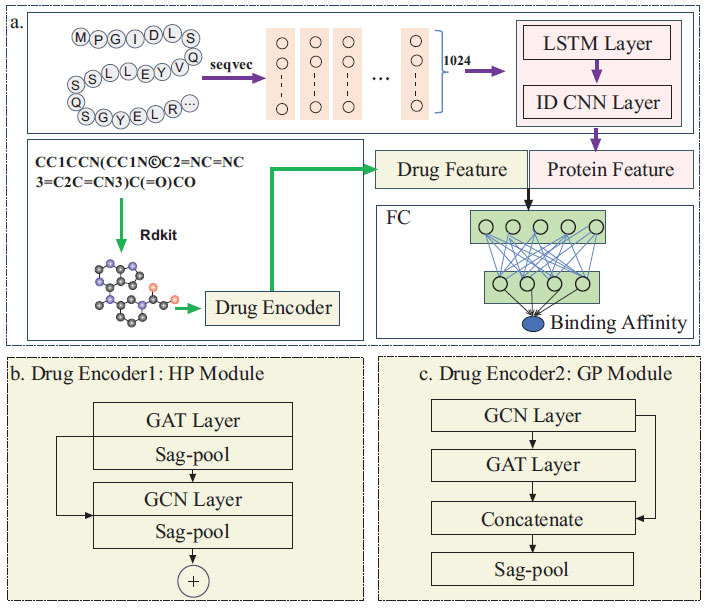
Overview of the LSTM-SAGDTA framework. (**a**) The input protein sequence is represented by a 1024-D vector by SeqVec. Subsequently, the protein feature is extracted by the LSTM and 1D-convolutional neural network. The drug molecule, meanwhile, is encoded to drug features by graph convolutional networks. The joint representation is input into a fully connected neural network to predict the DTI. (**b**) The drug encoder module 1, HierPool architectures, involves two blocks, which consist of a graph convolution layer and a SAGPooling layer. Finally, the two read-out results are summed. (**c**) The drug encoder module 2, GlobPool architecture, involves linking two graph convolution layers in series, combing the two outputs, and feeding them to the SAGPooling layer.

**Table 1:** Summary of the Davis, KIBA, and PDBbind datasets.

**Dataset**	**Proteins**	**Compounds**	**Interactions**
Davis	442	68	30056
KIBA	229	2111	118254
PDBbind	1606	4231	5041

**Table 2:** Atom features.

**Feature**	**Description**	**Size**
Atom type	C, N, O, S, F, Si, P, Cl, Br, Mg, Na, Ca, Fe, As, Al, I, B, V, K, TI, Yb, Sb, Sn, Ag, Pd, Co, Se, Ti, Zn, H, Li, Ge, Cu, Au, Ni, Cd, In, Mn, Zr, Cr, Pt, Hg, Pb, or “Unknown” (one-hot)	44
Num of H	Number of H bound to the atom	11
Degree	Number of directly bonded neighbors (one-hot)	11
Valence	Number of implicit H bound to the atom	11
Aromatic	Whether the atom is aromatic	1
Total	-	78

**Table 3:** The 5-fold cross-validation performance of LSTM-SAGDTA with HierPool.

**Dataset**	**Fold**	**MSE**	**CI**
Davis	1	0.219	0.895
	2	0.214	0.894
	3	0.205	0.894
	4	0.208	0.900
	5	0.214	0.892
	Average	0.212	0.895
Kiba	1	0.135	0.896
	2	0.139	0.895
	3	0.142	0.895
	4	0.153	0.894
	5	0.135	0.896
	Average	0.141	0.895

**Table 4:** The MSE and CI values for our methods compared to the baseline models on the Davis and Kiba datasets.

**Models**	**Davis**		**Kiba**	
**MSE**	**CI**	**MSE**	**CI**
KronRLS [15]	0.379	0.871	0.411	0.782
SimBoost [16]	0.282	0.872	0.222	0.836
DeepDTA [19]	0.261	0.878	0.194	0.863
WideDTA [21]	0.262	0.886	0.179	0.875
AttentionDTA [43]	0.245	0.887	0.162	0.882
DeepGS [38]	0.252	0.880	0.193	0.860
GraphDTA [34]	0.229	0.893	0.139	0.891
BiComp-DTA [28]	0.237	0.904	0.167	0.891
**LSTM-SAGDTA (HierPool)**	**0.206**	**0.903**	**0.125**	**0.901**
**LSTM-SAGDTA (GlobPool)**	**0.208**	**0.904**	**0.122**	**0.902**

**Table 5:** Performance comparison on the PDBbind dataset.

**Models**	**MSE**	**CI**
DeepDTA [19]	2.172	0.738
GraphDTA [34]	2.341	0.736
FusionDTA [73]	2.096	0.751
BiComp-DTA [28]	1.983	0.756
**Lstm-sagDTA (HierPool)**	**2.012**	**0.753**
**Lstm-sagDTA (GlobPool)**	**1.741**	**0.780**

**Table 6:** Result of comparison with 3-GCNs and GAT-GCN network architectures.

**Dataset**	**Model**	**MSE**	**CI**
Davis	3GCN (HierPool)	0.208	0.901
	GAT-GCN (HierPool)	0.206	0.903
	3GCN (GlobPool)	0.211	0.903
	**GAT-GCN (GlobPool)**	**0.208**	**0.904**
Kiba	3GCN (HierPool)	0.129	0.893
	GAT-GCN (HierPool)	0.125	0.901
	3GCN (GlobPool)	0.125	0.896
	**GAT-GCN (GlobPool)**	**0.122**	**0.902**

**Table 7:** Comparison of performances on different strategies.

**Dataset**	**Pooling Strategy**	**MSE**	**CI**
Davis	HierPool-max	0.212	0.901
	HierPool-mean	0.210	0.899
	**HierPool-SAG**	**0.206**	**0.903**
	GlobPool-max	0.208	0.903
	GlobPool-mean	0.211	0.900
	**GlobPool-SAG**	**0.208**	**0.904**
Kiba	HierPool-max	0.127	0.896
	HierPool-mean	0.125	0.900
	**HierPool-SAG**	**0.125**	**0.901**
	GlobPool-max	0.129	0.897
	GlobPool-mean	0.130	0.897
	**GlobPool-SAG**	**0.122**	**0.902**

**Note**: HierPool-max and GlobPool-max both utilize the Max-pooling layer, while HierPool-mean and GlobPool-mean both use the mean-pooling layer.

## Data Availability

The PyTorch-based source code and all data that were used in this study are available at https://github.com/blah957/blah957-lstm-sagDTA.

## LIST OF ABBREVIATIONS

CADD: Computer-aided Drug Design
CNN: Convolutional Neural Network
DTI: Drug-target Interactions
GAT: Graph Attention Networks
GCN: Graph Convolutional Networks
GIN: Graph Isomorphic Networks
GNN: Graph Neural Networks
HTS: High-throughput Screening
PDB: Protein Data Bank
